# Supplementary material for: Mulberry MnGolS2 Mediates Resistance to Botrytis cinerea on Transgenic Plants
Source: Genes (Basel). 2023 Oct 6;14(10):1912. doi: 10.3390/genes14101912 (PMC10606925; doi:10.3390/genes14101912)
Supplement: Supplementary file 1 [file genes-14-01912-s001.zip › genes-2581585-supplementary.pdf]

Table S1. Primers for PCR.

Gene symbol	Forward primer	Reverse primer
MnGolS2	TTTGGAGAGGACAGGGTACCATGGCTCCTGATATCATCA	CTCATTAAAGCAGGGAATTCCTAAGCCGCCGACGGGGC
MnGolS2 DL	AGTATTCGGCAACATAGACC	GAAGTAGAGAGGAGGTTTGG
AtBG2	CGTTGGAAATGAGGTGAAAC	TATCACTGGTTCGAGAAAGC
MnActin	GCATGAAGATCAAGGTGGTG	CATCTGCTGGAAGGTGCTAA
AtPR1	GCAGAACAACTAAGAGGCAA	CAGCGTAGTTGTAGTTAGCC
AtActin	TGCTGAGCTTATCGATTCCG	TTCGGTGATGGGAATACAG
